# Floristic survey of herbaceous and subshrubby aquatic and palustrine angiosperms of Viruá National Park, Roraima, Brazil

**DOI:** 10.3897/phytokeys.58.5178

**Published:** 2016-01-12

**Authors:** Suzana Maria Costa, Tiago Domingos Mouzinho Barbosa, Volker Bittrich, Maria do Carmo Estanislau do Amaral

**Affiliations:** 1 University of Campinas, Institute of Biology, Department of Plant Biology, P.O. Box 6109, 13083-970, Campinas, SP, Brazil; 2 R. Mário de Nucci, 500, Cidade Universitária, 13083-290, Campinas, SP, Brazil

**Keywords:** “Campinaranas”, Guiana Shield, flora, aquatic macrophytes, “Campinaranas”, Escudo das Guianas, flora, macrófitas aquáticas

## Abstract

We provide and discuss a floristic survey of herbaceous and subshrubby aquatic and palustrine angiosperms of Viruá National Park (VNP). The VNP is located in the northern Amazon basin and displays phytophysiognomies distributed in a mosaic where these plants occur, as flooded forests, hydromorphic white-sand savannas, “*buritizais*” and waterbodies. After expeditions between February/2010 and January/2015 and the analysis of specimens from regional herbaria, we list 207 species of herbaceous and subshrubby aquatic and palustrine angiosperms for the VNP, distributed in 85 genera in 37 families. We recorded six new occurrences for Brazil, two for the northern Brazilian region and 21 for Roraima state. These new occurrences, added to the other species listed here, highlight the floristic similarity between the study site and the Guiana Shield, an adjacent phytogeographical unit and geologically related to the origin of white-sand savannas.

## Abstract (pt)

(Lista Florística de angiospermas herbáceas e subarbustivas aquáticas e palustres do Parque Nacional do Viruá, Roraima, Brasil) Esse estudo foi realizado objetivando a elaboração de uma lista florística das angiospermas herbáceas e subarbustivas aquáticas e palustres do Parque Nacional do Viruá (PNV). O PNV está localizado na região norte da Bacia Amazônica e apresenta fitofisionomias distribuídas em mosaico onde esse grupo de plantas ocorre, como florestas inundáveis, savanas sobre solos de areia branca hidromórficas, buritizais e corpos d’água. Após coletas entre Fevereiro/2010 e Janeiro/2015 e a análise de espécimes depositados em herbários regionais, foram listadas 207 espécies de angiospermas herbáceas e subarbustivas aquáticas e palustres no PNV, distribuídas em 85 gêneros e 37 famílias. Foram registradas seis novas ocorrências para o Brasil, duas para a região norte do Brasil e 21 para o estado de Roraima. Essas novas ocorrências, juntamente com as demais espécies listadas, evidenciam a similaridade florística entre a área de estudo e o Escudo das Guianas, uma unidade fitogeográfica adjacente e geologicamente relacionada com a origem das savanas sobre solos de areia branca.

## Introduction

Aquatic and palustrine (A&P) plants are able to survive in permanent or periodic submersion of at least their root system and share a few of the adaptations to these habitats (Sculthorpe 1967, Philbrick and Les 1996, Amaral et al. 2008). These plants form an artificial group that includes bryophytes, ferns and angiosperms (Sculthorpe 1967, Chambers et al. 2008) and contains species with pronounced phenotypic plasticity (Sculthorpe 1967) which hinder their identification. The use of several bibliographic resources and the detailed examination of specimens are indispensable for a reasonably reliable identification. Concurrently, there are a number of difficulties and peculiarities related to the collecting and preservation process (Fidalgo and Bononi 1989) such as the need of boats, recipients and special papers to press the plants correctly.

Aquatic and palustrine species are important for the structure and maintenance of the habitats where they occur. These plants determine the environmental heterogeneity and water quality of natural and artificial waterbodies (Junk 1986, Cronk and Fenessy 2001). Studies on A&P plants in the Neotropics focus mainly on ecological analyses, while floristic and taxonomic analyses are sparse (Padial et al. 2008, Piedade et al. 2010).

The Amazon region contains complex river systems with different physicochemical characteristics resulting in two contrasting types of inundated forests, one known as *várzea* – along white-waters rivers rich in nutrients and suspended sediment – and the other as *igapó* – along rivers poor in nutrients and, generally, poor in suspended sediments with dark or clear waters (Pires and Prance 1985). These distinctions arise from the origin and drainage areas of rainwater and directly influence the diversity of plants, particularly A&P ones. According to Piedade et al. (2010), studies focusing on the richness and ecology of wetland plants are more common in areas of *várzea* and inventories are still needed in *igapó* areas.

The Viruá National Park (VNP) is among the few protected areas that preserves ecosystems favorable to wetland communities. It receives water discharges from different rivers, of different sizes and mostly with *igapó* characteristics (Junk et al. 2011). The distribution of the vegetation in the VNP shows a mosaic-like organization with large areas where the soil is permanently or periodically submersed or saturated with water (mainly white-sand savannas, locally known as “*campinaranas*”) (ICMBio 2014). The white-sand savannas can vary from a forested to herbaceous physiognomy (Veloso et al. 1991); this gradual change may be associated with the increasing waterlogging of the soils (Mendonça 2011). The herbaceous physiognomy of white-sand savannas covers about 25% of the VNP (ICMBio 2014).

Reports of some preliminary studies in the VNP mention a high floristic richness (Gribel et al., unpublished data) but unfortunately, if there are vouchers from these expeditions, none are in any herbaria known to us. Additionally, Gribel et al. (unpublished data) rarely identified herbs and subshrubs at species level, and often listed wetland plants by popular names or only at family or genus level. In fact, these authors never published formal checklists or indicated the material identified during their inventories.

Keeping in mind the existence of vast areas of periodically or permanently inundated ecosystems in the Viruá National Park and the lack of knowledge relative to wetland plants in the region and in areas influenced by *igapó* rivers, we provide and discuss the floristic survey of herbaceous and subshrubby aquatic and palustrine angiosperms found there.

## Methods

### Study area

**Figure 1.**
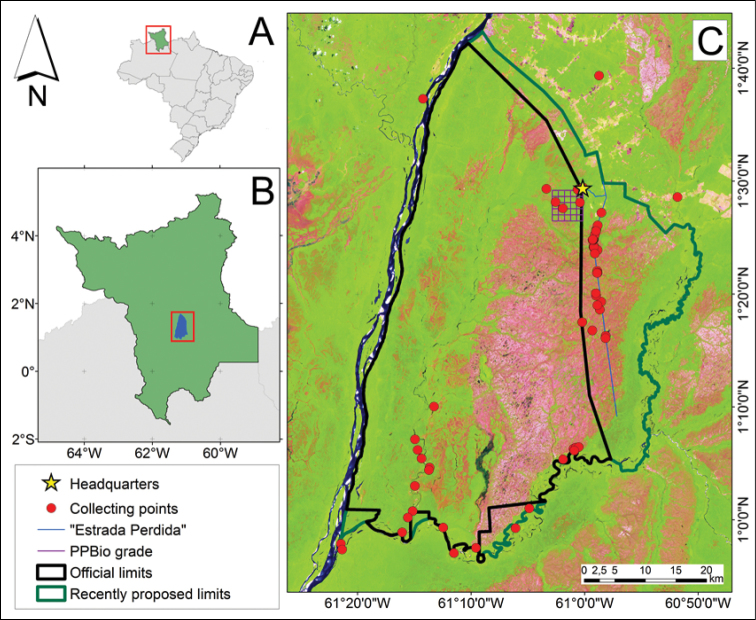
Viruá National Park (location). **A** Roraima state in Brazil **B** the VNP in the central-southern region of Roraima **C** the actual limits of protected area (black line), the area aimed to be included during extension (green line) and the collecting points [.shp files provided by IBGE and the VNP administration]

The Viruá National Park (VNP; Figure 1) is located in the Caracaraí district, Roraima state, northern Brazil (1°19'11"N; 61°7'17"W DMS). The climate in the region is equatorial with the rainy season intercalated by a more or less short dry season, between October and March (ICMBio 2014). This protected area presents igneous volcanic or metamorphic rocks in the hills and sandy soil of fluvial, aeolian or weathering sedimentary origin in the plains (ICMBio 2014).

**Figure 2.**
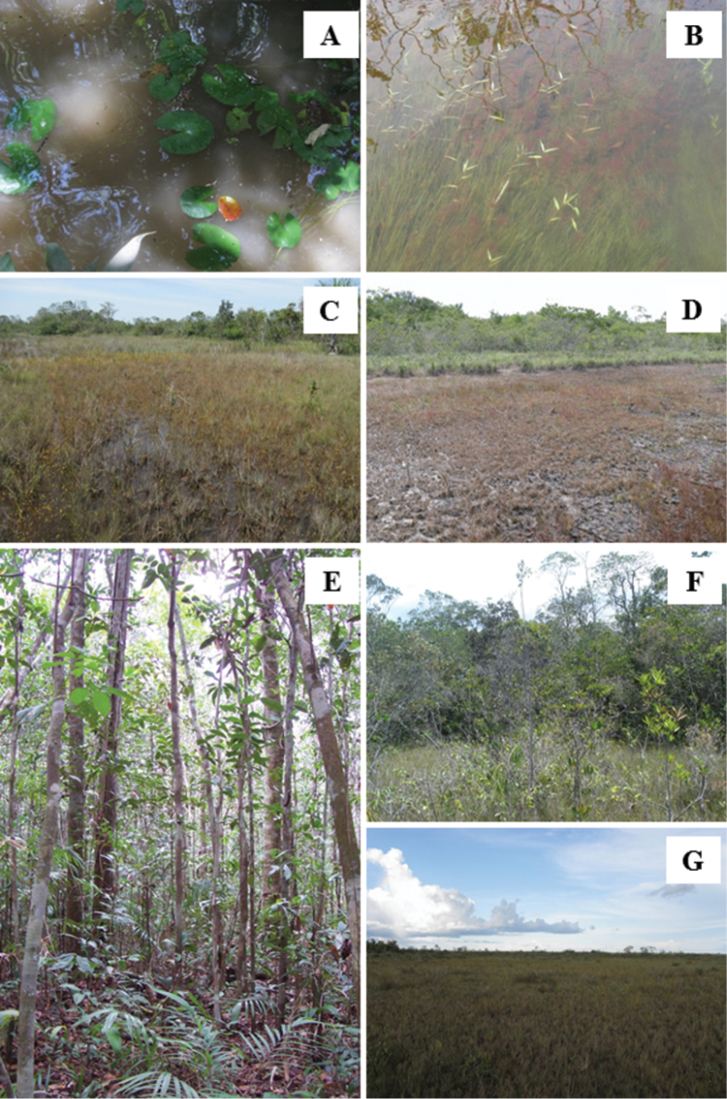
Viruá National Park: habitats and physiognomies. **A–B** waterbodies with turbid (**A**) and translucid (**B**) water; **C–D** Areas with saturated soils during rainy season (**C**) and dry season (**D**); **E–G** Forested (**E** given by K.G. Cangani), arboreal (**F**) and herbaceous (**G**) white-sand savannas (“campinaranas”).

The VNP contains in its 227,011 ha different plant formations distributed in a mosaic (ICMBio 2014) (Figures 1–2): rainforest – typical forested formation of amazon region; white-sand savannas- sandy and leached, forested to grassy, hydromorphic or non-hydromorphic plain areas; and “buritizais” – flooded areas dominated by *Mauritia* palms. The protected area has its western boundary at the Branco River, a line drawn a few kilometers from an abandoned fragment of the BR-174 road (known as “Estrada Perdida”) as the northern and the eastern boundaries, and by the Anauá River in the southern limit. In addition to the water discharges received from rivers mentioned above, it also receives water from the Barauana River, situated to the east and beyond the limits of the VNP, from the Iruá River, in a south-north axis, and a dense network of streams within its boundaries (ICMBio 2014).

### Collecting and analyzing data

We investigate the herbaceous and subshrubby aquatic and palustrine angiosperms. The expeditions to collect fertile botanical samples encompassed the local dry and wet seasons, between February/2010 and January/2015. We followed Fidalgo and Bononi (1989) for both the collecting and the herborization processes and vouchers are deposited mainly at INPA and UEC herbaria. When available, we sent duplicates to UFP and/or UFRR herbaria. The acronyms are according to Index Herbariorum (Thiers 2015, continuously updated). Our inventory also included specimens previously collected in the study area and deposited at INPA, MIRR and UFRR herbaria.

We chose the collecting points non-systematically but they tended to be more concentrated in peripheral areas and along “Estrada Perdida”, due to their accessibility, and in areas where aquatic and palustrine plants are abundant (white-sand savannas of hydromorphic soils, “buritizais” and waterbodies) (Figure 1). Even though the “Estrada Perdida” is outside of the current protected area, a proposal suggests the enlargement of VNP’s borders aiming for the inclusion of areas eastwards up to the margins of the Barauana River, such that the “Estrada Perdida” would be enclosed in the VNP (ICMBio 2014).

Our identifications were based on regional floras of the Amazon and Guiana regions and specialized bibliography of each family: Core 1936, Organization for Flora Neotropica 1967- , Cook 1985, Cook and Urmi-König 1985, Kral 1988, 1992, Taylor 1989, 1991, Thomas 1992, 1997, Steyermark et al. 1995 – 2005, Araújo and Longhi-Wagner 1996, Camelbeke et al. 1997, Luceño et al. 1997, Kral and Jansen-Jacobs 1998, Wanderley et al. 2001-, Prata 2002, Gil and Bove 2004, Campbell 2005, Trevisan 2005, Simpson 2006, Thomas 2006, Rivadavia et al. 2009, Souza and Giulietti 2009, Wanderley 2011, Christenhusz 2014.

Additionally, we studied images of specimens deposited at F, K, MO, NY, P, and other herbaria with online digital images. We took into account the reliability of the identifications, with preference to types, historical collections and specimens identified by specialists; when available in the literature, we queried the original descriptions and revisions of genera or entire families. Moreover, we consulted specialists when necessary.

Rivadavia et al. (2009) described *Drosera amazonica* Rivadavia, A. Fleischm. & Vicent. and cited the VNP among the localities of its occurrence, and although we did not collect this species, it appears in our list. Pessoa et al. (2015) and Mota et al. (2015), respectively, held two taxonomic treatments with focus on Orchidaceae and Xyridaceae to this area, and we also included the aquatic and palustrine herbs and subshrubs cited by them in our list. When it was impossible to examine directly any specimen of the species treated, we cited the correspondent treatment.

Information on geographic distribution and authors of the species were based on TROPICOS (last access sept/2012), “Lista de Espécies da Flora do Brasil” (Forzza et al. 2015) and specialized literature for each family.

We classified the species analyzed according to morphoanatomical characters and indicated the functional ecological group that they belong (“life forms”). The categories represent a continuum from less to more specialized adaptations to the aquatic environment: palustrine plants (growing in saturated soils); emergent plants; rooted with floating leaves; free floating; and submersed plants (with life cycle entirely or partially under water) (Cronk and Fenessy 2001, Chambers et al. 2008).

For floristic comparisons, we unite some representative lists from northern Brazil and other adjacent floras, namely: savannas from northern Roraima (Miranda and Absy 1997), *Várzea* and *Igapó* areas (Piedade et al. 2010, Lopes et al. 2014; Junk and Piedade 1993, Conserva et al. 2008), Guiana Shield (Funk et al. 2007), and Northern Brazil (Moura-Júnior et al. 2015). We made an overall comparison between the lists and no richness or diversity index was estimated (Table 1).

**Table 1.** Aquatic and palustrine herbaceous and subshrubby angiosperms of the Viruá National Park [in brackets the number of genera and species of each family; (*) = new occurrences for Roraima state; (#) = new occurrences for northern Brazil; (&) = new occurrences for Brazil; (¥) = commercialization incompatible with species survival according to UNEP World Conservations Monitoring Centre; ($)= in Brazil, occurs only in Roraima; pal = palustrine; emer = emergent; fleav = with floating leaves; ffloat = free floating; sub = submerged. When the accepted names are different in the TROPICOS database and in Forzza et al. (2015), the name cited by the latter is listed in brackets; (^+^) = probably endemic to white-sand savannas; F=Funk et al. 2007; C = Conserva et al. 2008; P = Piedade et al. 2010, MJr = Moura Junior et al. 2015].

SPECIES	Life form	Occurrence in Brazil	Also listed at	Selected Vouchers
ALISMATACEAE (2/4)				
*Helanthium tenellum* (Martius) Britton	pal, emer, sub	???	F, MJr	TDM Barbosa 1094 (INPA, UEC)
*Sagittaria guayanensis* Kunth	emer, fleav	N, NE, CO, SE	M&A, F, MJr	TDM Barbosa 1097 (UEC)
*Sagittaria rhombifolia* Cham.	emer	N, NE, CO, SE, S	M&A, F, MJr	TDM Barbosa 1110 (INPA, UEC)
APOCYNACEAE (1/3)				
*Cynanchum guanchezii* Morillo	pal	N, NE	F	SM Costa 836 (INPA, UEC)
*Cynanchum sobradoi* Morillo [=*Ditassa sobradoi* (Morillo) Liede]	pal	N	F	SM Costa 865 (UEC); TDM Barbosa 1241 (INPA)
*Cynanchum strictum* (Gleason & Moldenke) R.W.Holm * [=*Tassadia stricta* (E.Fourn.) Liede & Rapini]	pal	N	F	SM Costa 994 (INPA, UEC)
ARACEAE (2/2)				
*Montrichardia arborescens* (L.) Schott	pal, emer	N, NE, CO, SE	M&A, J&P, C, F, MJr	TDM Barbosa 768 (INPA, UEC)
*Pistia stratiotes* L.	ffloat	N, NE, CO, SE, S	J&P, C, F, MJr	TDM Barbosa 1403 (UEC)
ARECACEAE (1/1)				
*Bactris campestris* Poepp.	pal	N, NE	F	MJG Hopkins 2181 (INPA), SM Costa 840 (UEC);
ASTERACEAE (1/1)				
Eclipta aff. alba (L.) Hassk.	pal	N, NE, CO, SE, S	J&P, F, MJr	SM Costa 968 (INPA, UEC)
BROMELIACEAE (1/1)				
Ananas cf. parguazensis Camarco & L.B.Sm.	pal	N	F	TDM Barbosa 1438 (INPA, UEC)
BURMANNIACEAE (1/2)				
*Burmannia bicolor* Mart.	pal	N, NE, CO, SE, S	F	SM Costa 870 (UEC), R Goldenberg 1606 (INPA)
*Burmannia capitata* (Walter ex J.F.Gmel.) Mart.	pal	N, NE, CO, SE, S	F	SM Costa 792 (INPA, UEC)
CABOMBACEAE (1/2)				
*Cabomba schwartzii* Rataj [= *Cabomba aquatica* Aubl.]	sub, fleav	N, NE, SE, S	F, P?, MJr?	TDM Barbosa 1230 (INPA, UEC)
*Cabomba furcata* Schult. & Schult. f.	sub, fleav	N, NE, CO, SE, S	F, MJr	TDM Barbosa 1201 (INPA, UEC)
CYPERACEAE (14-15/45)				
*Bulbostylis conifera* (Kunth) C.B. Clarke	pal	N, NE, CO, SE, S	M&A, F	TDM Barbosa 1392 (INPA, UEC)
*Bulbostylis junciformis* (Kunth) C.B.Clarke	pal	N, NE, CO, SE, S	M&A, F	TDM Barbosa 1115 (INPA, UEC)
*Bulbostylis lanata* (Kunth) Lindm.	pal	N, NE	M&A, F	TDM Barbosa 1331 (INPA, UEC)
*Calyptrocarya monocephala* Hochst. ex Steud. ^$^	pal	N	F	TDM Barbosa 1294 (INPA, UEC)
*Cyperus aggregatus* (Willd.) Endl.	pal	N, NE, CO, SE, S	M&A, F, MJr	TDM Barbosa 1113 (INPA, UEC)
*Cyperus haspan* L.	pal, emer	N, NE, CO, SE, S	M&A, J&P, F, MJr	TDM Barbosa 1104 (INPA, UEC)
*Cyperus simplex* Kunth	pal	N, NE, CO, SE	F	TDM Barbosa 1413 (INPA, UEC)
*Cyperus surinamensis* Rottb.	pal	N, NE, CO, SE, S	M&A, J&P, C, F, MJr	TDM Barbosa 1112 (INPA, UEC)
*Cyperus* sp.	pal	-		TDM Barbosa 1404 (INPA, UEC)
Diplacrum cf. capitatum (Willd.) Boeckeler	pal, emer	N, NE, CO	F	TDM Barbosa 1126 (INPA, UEC)
*Diplacrum guianense* (Nees) T.Koyama	pal, emer	N, CO	F	SM Costa 924 (INPA)
*Eleocharis acutangula* (Roxb.) Schult.	pal, emer	N, NE, CO, SE, S	F	TDM Barbosa 1195 (UEC)
*Eleocharis fluctuans* (L.T. Eiten) E.H. Roalson & C.E. Hinchliff	sub	N	F	TDM Barbosa 1237 (INPA)
*Eleocharis geniculata* (L.) Roem. & Schult.	pal, emer	N, NE, CO, SE, S	C, F, MJr	EM Pessoa 790 (INPA)
*Eleocharis interstincta* (Vahl) Roem. & Schult.	pal, emer	N, NE, CO, SE, S	M&A, C, F, MJr	TDM Barbosa 1298 (INPA, UEC)
*Eleocharis* sp	sub	-		
*Exochogyne amazonica* C.B. Clarke	pal	N, NE, CO, SE	F	SM Costa 737 (INPA, UEC)
*Fimbristylis vahlii* (Lam.) Link.	pal	N, NE	F	SM Costa 1005 (INPA)
*Fuirena umbellata* Rottb.	pal, emer	N, NE, CO, SE, S	M&A, C, F, MJr	TDM Barbosa 1253 (INPA, UEC)
*Hypolytrum pulchrum* (Rudge) H. Pfeiff.	pal	N, NE	F	TDM Barbosa 1164 (INPA,UEC)
*Lagenocarpus celiae* T. Koyama & Maguire ^+^	pal	N	F	TDMB Barbosa 1263 (INPA, UEC)
*Lagenocarpus eriopodus* T.Koyama & Maguire * ^+^	pal, emer	N	F	SM Costa 1167
*Lagenocarpus glomerulatus* Gilly	pal	N	F	SM Costa 793 (INPA, UEC)
*Lagenocarpus rigidus* (Kunth) Nees	pal	N, NE, CO, SE, S	M&A, F	SM Costa 938 (INPA, UEC)
*Lagenocarpus sabanensis* Gilly	pal	N	F	FRC Costa 1650 (INPA)
*Lagenocarpus verticillatus* (Spreng.) T.Koyama & Maguire [=*Cryptangium verticillatum* (Spreng.) Vitta]	pal	N, NE, CO, SE	F	SM Costa 803 (INPA, UEC)
*Oxycaryum cubense* (Poepp. & Kunth) Lye	emer	N, NE, CO, SE, S	C, F, MJr	SM Costa 971 (INPA, UEC)
*Pycreus polystachyos* (Rottb.) P.Beauv.	pal	N, NE, CO, SE, S	F, MJr	SM Costa 1004 (INPA), TDM Barbosa 1075 (UEC)
*Rhynchospora barbata* (Vahl) Kunth	pal	N, NE, CO, SE	M&A, F, MJr	TDM Barbosa 1328 (INPA, UEC)
*Rhynchospora cephalotes* (L.) Vahl	pal	N, NE, CO, SE	M&A, F	TDM Barbosa 1189 (INPA, UEC)
*Rhynchospora emaciata* (Nees) Boeck.	pal	N, NE, CO, SE, S	M&A, F, MJr	TDM Barbosa 1127 (INPA)
*Rhynchospora globosa* (Kunth) Roem. & Schult.	pal, emer	N, NE, CO, SE, S	M&A, F, MJr	SM Costa 988 (INPA, UEC)
*Rhynchospora hirsuta* (Vahl) Vahl	pal	N, NE, CO	M&A, F	SM Costa 733 (INPA, UEC)
*Rhynchospora holoschoenoides* (Rich.) Herter	pal, emer	N, NE, CO, SE, S	M&A, F, MJr	TDM Barbosa 1076 (INPA, UEC)
*Rhynchospora longibracteata* Boeck.	pal, emer	N	F	SM Costa 864 (INPA,UEC)
*Rhynchospora maguireana* T. Koyama^&^	pal, emer	-	F	TDM Barbosa 1169 (INPA, UEC)
*Rhynchospora riparia* (Nees) Boeck.	pal	N, NE, CO, SE, S	F	TDM Barbosa 1123 (INPA)
*Rhynchospora rugosa* (Vahl) Gale	pal	N, NE, CO, SE, S	F	TDM Barbosa 1278 (INPA, UEC)
*Rhynchospora schomburgkiana* (Boeck.) T. Koyama	pal	N	F, MJr	TDM Barbosa 1432 (INPA, UEC)
*Rhynchospora trichochaeta* C.B.Clarke *	pal	N, NE, CO	F	TDM Barbosa 1326 (INPA, UEC)
*Rhynchospora trispicata* (Nees) Schrad.	pal, emer	N, NE, CO	F	TDM Barbosa 1254 (INPA, UEC)
*Scleria amazonica* Camelbeke, M.Strong & Goetgh.^&^	pal, emer	-	F	TDM Barbosa 1259 (INPA, UEC)
*Scleria cyperina* Kunth	pal	N, NE, CO, SE	F	SM Costa 890 (INPA, UEC)
Scleria cf. lacustris C.Wright	emer	N	F	TDM Barbosa 1267 (INPA, UEC)
*Scleria reticularis* Michx.	pal	N, NE		SM Costa 720 (INPA, UEC)
DROSERACEAE (1/2)				
*Drosera amazonica* Rivadavia, A. Fleischm. & Vicent.	pal	N (*vide* Rivadavia et al. 2009)	-	(vide Rivadavia et al. 2009)
*Drosera kaieteurensis* Brumm.-Ding. ^&^	pal		F	TDM Barbosa 1284 (INPA, UEC)
ERIOCAULACEAE (4/15)				
*Eriocaulon setaceum* L.	sub	N, CO, SE		TDM Barbosa 1219 (UEC)
*Eriocaulon tenuifolium* Klotzsch ex Körn. ^$^	pal, emer, sub	N	F	TDM Barbosa 1051 (UEC)
*Paepalanthus tortilis* (Bong.) Mart.	pal	N, NE, SE	F	TDM Barbosa 1064
*Syngonanthus anomalus* (Körn.) Ruhland	sub	N	F, MJr	MCE Amaral 2011/17 (UEC)
*Syngonanthus caulescens* (Poir.) Ruhland	pal	N, NE, CO, SE, S	F, MJr	SM Costa 726 (UEC)
*Syngonanthus cuyabensis* (Bong.) Giul., Hensold & L.R. Parra*	pal	N, NE, CO, SE	F	FN Cabral 501 (UEC)
*Syngonanthus fenestratus* Hensold	pal	N	F	MCE Amaral 2011/26 (UEC)
*Syngonanthus gracilis* (Bong.) Ruhland	pal	N, NE, CO, SE, S	F, MJr	SM Costa 702 (UEC)
*Syngonanthus humboldtii* (Kunth) Ruhland	pal	N, NE, CO	F	TDM Barbosa 1046 (UEC)
*Syngonanthus longipes* Gleason	pal	N, CO	F	SM Costa 787 (UEC)
*Syngonanthus spongiosus* Hensold	pal	N		MCE Amaral 2011/5 (UEC)
*Syngonanthus tenuis* (Kunth) Ruhland	pal	N, CO	F	MCE Amaral 2011/29 (UEC), TDM Barbosa 1220 (INPA)
*Syngonanthus trichophyllus* Moldenke	pal	N	F	TDM Barbosa 1321 (UEC)
*Syngonanthus umbellatus* (Lam.) Ruhland	pal	N, CO, SE	F	SM Costa 736 (INPA, UEC)
*Tonina fluviatilis* Aubl.	pal	N, NE, SE	F, MJr	TDM Barbosa 1066 (INPA, UEC)
EUPHORBIACEAE (1/1)				
*Croton subserratus* Jabl. ^&^	emer	-		TDM Barbosa 1080 (UEC)
GENTIANACEAE (3/4)				
*Chelonanthus alatus* (Aubl.) Pulle	pal	N, CO	F	TDM Barbosa 1059 (INPA)
*Coutoubea reflexa* Benth.	pal	N	F	TDM Barbosa 1139 (INPA, UEC)
*Irlbachia pratensis* (Kunth) L.Cobb & Maas ^+^	pal, emer	N	F	SM Costa 932 (INPA, UEC)
*Irlbachia pumila* (Benth.) Maguire *	pal	N	F	TDM Barbosa 1067 (UEC)
HAEMODORACEAE (1/1)				
*Schiekia orinocensis* (Kunth) Meisn.	pal	N, NE, CO	M&A, F, MJr	TDM Barbosa 1194 (UEC)
HYDROCHARITACEAE (1/1)				
*Elodea granatensis* Bonpl. [=*Apalanthe granatensis* (Bonpl.) Planch.]	sub	N, NE, CO, SE	F, MJr	TDM Barbosa 1323 (INPA)
LEGUMINOSAE (2/2)				
*Aeschynomene scabra* G.Don^#^	pal, emer	NE	F	MCE Amaral 2011/13 (UEC)
*Zornia latifolia* Sm.	pal	N, NE, CO, SE, S	F, MJr	TDM Barbosa 1118 (INPA, UEC)
LENTIBULARIACEAE (2/25)				
*Genlisea filiformis* A.St.- Hil.	pal	N, NE, CO, SE	F	SM Costa 715b (INPA, UEC)
*Genlisea oxycentron* A.St.-Hil.*	pal	N, NE		SM Costa 715a (INPA, UEC)
*Genlisea pygmaea* A.St.- Hil.	pal	N, NE, CO, SE	F	SM Costa 698 (INPA, UEC)
*Utricularia amethystina* Salzm. ex A. St.-Hil. & Girard	pal	N, NE, CO, SE	F	SM Costa 695 (INPA, UEC)
*Utricularia benjaminiana* Oliv. ^$^	sub	N	F	TDM Barbosa 1106 (INPA, UEC)
*Utricularia breviscapa* Wright ex Griseb.	sub	N, NE, CO, SE	F, MJr	SM Costa 858 (INPA, UEC)
*Utricularia chiribiquetensis* Fernandez-Pérez*	pal	N	F	SM Costa 779 (INPA, UEC)
*Utricularia costata* P. Taylor	pal	N, NE, CO	F	TDM Barbosa 1320 (INPA, UEC)
*Utricularia cucculata* A.St.-Hil. & Girard	sub	N, NE, CO, SE, S	F	SM Costa 712 (INPA, UEC)
*Utricularia foliosa* L.	sub	N, NE, CO, SE, S	M&A, J&P, P, C, F, MJr	SM Costa 767 (INPA, UEC)
*Utricularia gibba* L.*	sub	N, NE, CO, SE, S	M&A, J&P, C, F, MJr	SM Costa 895 (INPA, UEC)
*Utricularia guyanensis* A.DC.*	pal	N, NO, CO	M&A, F, MJr	SM Costa 756 (INPA, UEC)
*Utricularia hispida* Lam.*	pal	N, NE, CO, SE	F	SM Costa 869 (INPA, UEC)
*Utricularia hydrocarpa* Vahl*	sub	N, NE, CO, SE, S	F	SM Costa 744 (INPA, UEC)
*Utricularia juncea* Vahl	pal	N, NE	F	SMCosta 746 (INPA, UEC)
*Utricularia longeciliata* DC.	pal	N	F	TDM Barbosa 1345 (INPA, UEC)
*Utricularia myriocista* A.St.-Hil. & Girard	sub	N, NE, CO, SE	M&A, C, F, MJr	SM Costa 740 (INPA, UEC)
*Utricularia nana* A.St.-Hil. & Girard*	pal	N, NE, CO, SE, S	F	SM Costa 699 (INPA, UEC)
*Utricularia olivacea* Wright ex. Girard*	sub	N, CO, SE, S	F	SM Costa 727 (INPA, UEC)
*Utricularia pusilla* Vahl	pal	N, NE, CO, SE	M&A, F, MJr	SM Costa 691 (INPA, UEC)
*Utricularia sandwithii* P. Taylor	pal	N	F	SM Costa 701 (INPA, UEC)
*Utricularia simulans* Pilg.	pal	N, NE, CO, SE	M&A, F, MJr	SM Costa 716 (INPA, UEC)
*Utricularia subulata* L.	pal	N, NE, CO, SE, S	M&A, F, MJr	SM Costa 706 (INPA, UEC)
*Utricularia triloba* Benj.	pal	N, NE, CO, SE, S	M&A, F, MJr	SM Costa 709 (INPA, UEC)
*Utricularia viscosa* Spruce ex Oliver	pal	N, NE, CO	F	SM Costa 719 (INPA, UEC)
LINDERNIACEAE (1/1)				
*Lindernia diffusa* (L.) Wettst.	pal	N, NE, CO, SE, S	F, MJr	TDM Barbosa 1250 (INPA, UEC)
LYTHRACEAE(1/1)				
Cuphea cf. gracilis Kunth^&^	pal		F	TDM Barbosa 1132 (UEC)
MAYACACEAE (1/2)				
*Mayaca fluviatilis* Aubl.	pal, sub	N, NE, CO, SE, S	F, MJr	SM Costa 873 (INPA, UEC)
*Mayaca longipes* Mart. ex Seub.	pal, sub	N, NE, CO, SE	F, MJr	TDM Barbosa 1099 (INPA, UEC)
MELASTOMATACEAE (7/10)				
*Acisanthera crassipes* (Naudin) Wurdack	pal, emer	N, NE, CO	F	MJR Rocha 747 (BHCB)
*Acisanthera tetraptera* (Cogn.) Gleason	pal, emer	N	F	TDM Barbosa 1386 (INPA)
*Comolia microphylla* Benth.	pal	N	F	TDM Barbosa 1111 (INPA, UEC)
*Comolia villosa* (Aubl.) Triana	pal	N, NE	F, MJr	TDM Barbosa 1182 (INPA)
*Macairea lasiophylla* (Benth.) Wurdack ^+^	pal	N	M&A, F	KG Cangani 160 (INPA)
*Pachyloma coriaceum* DC.	pal	N	F	R Goldenberg 1591a (INPA)
*Pachyloma huberioides* (Naudin) Triana	pal	N	F	TDM Barbosa 1205 (INPA, UEC)
*Rhynchanthera grandiflora* (Aubl.) DC.	pal	N, NE, CO, SE	F, MJr	MK Caddah 871 (INPA, UEC)
*Siphanthera cowanii* Wurdack ^& +^	pal		F	SM Costa 926 (INPA, UEC)
*Tibouchina aspera* Aubl.	pal	N, NE, CO	M&A, F, MJr	TDM Barbosa 1317 (INPA, UEC)
MENYANTHACEAE (1/1)				
*Nymphoides indica* (L.) Kuntze	fleav	N, NE, CO, SE, S	M&A, C, F, MJr	TDM Barbosa 1101 (UEC), GA Gomes-Costa 114 (INPA)
MOLLUGINACEAE (1/1)				
*Glinus radiatus* (Ruiz & Pav.) Rohr.	pal	N, NE, CO, SE, S	F	SM Costa 1063
NYMPHAEACEAE (1/3)				
*Nymphaea amazonum* Mart. & Zucc.*	fleav	N, NE, CO, SE, S	J&P, F	MCE Amaral 2015/19 (INPA)
*Nymphaea gardneriana* Planch.	fleav	N, NE, CO, SE, S	C, F, MJr	TDM Barbosa 1229 (UEC)
*Nymphaea rudgeana* G.Mey.	fleav	N, NE, SE, S	M&A, F, P, MJr	SM Costa 815 (UEC)
OCHNACEAE (1/3)				
*Sauvagesia erecta* L.	pal	N, NE, CO, SE, S	M&A, F, MJr	TDM Barbosa 1102 (UEC)
*Sauvagesia ramosa* (Gleason) Sastre	pal	N	F	TDM Barbosa 1335 (INPA, UEC)
*Sauvagesia sprengelii* A.St.-Hil.	pal	N, NE, SE	M&A, F, MJr	TDM Barbosa 1166 (INPA, UEC)
ONAGRACEAE (1/4)				
*Ludwigia hyssopifolia* (G.Don) Exell	pal, emer	N, NE, CO, SE, S	F, MJr	SM Costa 973 (INPA, UEC)
*Ludwigia leptocarpa* (Nutt.) H.Hara	pal, emer	N, NE, CO, SE, S	J&P, F, MJr	TDM Barbosa 1410 (INPA, UEC)
*Ludwigia nervosa* (Poir.) H.Hara	pal, emer	N, NE, CO, SE, S	M&A, F, MJr	TDM Barbosa 1234 (INPA, UEC)
*Ludwigia sedoides* (Humb. & Bonpl.) H.Hara	sub, fleav	N, NE, CO, SE	M&A, C, F, MJr	SM Costa 764 (INPA, UEC)
ORCHIDACEAE (8/10)				
*Catasetum discolor* (Lindley) Lindley	pal	N, NE, SE	(vide Pessoa et al. 2015)	(vide Pessoa et al. 2015)
*Cleistes rosea* Lindl.	pal, emer	N, NE, CO, SE, S	M&A, F	SM Costa 1184 (INPA, UEC)
*Cleistes tenuis* (Reichenbach f. ex Grisebach) Schlechter	pal	N, NE, CO, SE, S	(vide Pessoa et al. 2015)	(vide Pessoa et al. 2015)
*Duckeella pauciflora* Garay ^¥ $^	pal	N	F	TDM Barbosa 1424 (INPA, UEC)
*Epidendrum orchidiflorum* Salzmann ex Lindley	pal	N, NE, SE	(vide Pessoa et al. 2015)	(vide Pessoa et al. 2015)
*Epistephium lucidum* Cogn.	pal	N, NE, CO, SE	F	E Pessoa 742 (INPA)
*Epistephium parviflorum* Lindley	pal	N, CO	(vide Pessoa et al. 2015)	(vide Pessoa et al. 2015)
*Galeandra devoniana* M.R. Schomb. ex Lindl.	pal	N	F	TDM Barbosa 1270 (INPA, UEC)
*Habenaria schwackei* Barb. Rodr.	pal, emer	N, NE, CO, SE, S		TDM Barbosa 1309 (INPA, UEC)
*Nohawilliamsia pirarensis* (Reichenbach f.) M.W.Chase & Whitten	pal	N	(vide Pessoa et al. 2015)	(vide Pessoa et al. 2015)
OROBANCHACEAE (1/1)				
*Agalinis hispidula* (Mart.) D’Arcy	pal	N, NE, CO	F	FN Cabral 473 (UEC)
				
PLANTAGINACEAE (1/2)				
*Bacopa egensis* (Poepp.) Pennell*	pal, sub	N, CO	F, MJr	SM Costa 1061 (UEC)
*Bacopa reflexa* (Benth.) Edwall	sub	N, NE, CO	F, MJr	SM Costa 755 (INPA,UEC)
POACEAE (7/12)				
*Andropogon bicornis* L.	pal	N, NE, CO, SE, S	M&A, F, MJr	TDM Barbosa 1276 (UEC)
*Andropogon leucostachyus* Kunth	pal	N, NE, CO, SE, S	M&A, F	TDM Barbosa 1150 (UEC)
*Andropogon virgatus* Desv.*	pal	N, NE, CO, SE, S	M&A, F	TDM Barbosa 1261 (UEC)
*Axonopus fissifolius* (Raddi) Kuhlm.*	pal	N, NE, CO, SE, S	F	PL Viana 5207 (INPA)
*Axonopus pubivaginatus* Henr.	pal	N, NE, SE	F	TDM Barbosa 1072 (UEC)
*Echinolaena inflexa* (Poir.) Chase	pal, emer	N, NE, CO, SE, S	F	TDM Barbosa 1183 (UEC)
*Oryza rufipogon* Griff.*	emer	N, CO	F, P	TDM Barbosa 1231 (UEC)
*Otachyrium grandiflorum* Send. & Soderstr.	pal	N, CO	F	TDM Barbosa 1062 (UEC)
*Otachyrium versicolor* (Döll) Henrard	pal	N, NE, CO, SE, S	F	TDM Barbosa 1109 (UEC)
Paspalum cf. lacustre Chase ex Swallen*	emer	N	F	TDM Barbosa 1245 (UEC)
*Paspalum repens* P.B. Bergius	emer	N, NE, CO, SE, S	M&A, C, F, MJr	TDM Barbosa 1204 (UEC)
*Trichanthecium cyanescens* (Nees ex. Trin.) Zuloaga & Morrone	pal	N, NE, CO, SE, S	MJr	
POLYGALACEAE (1/5)				
*Polygala adenophora* DC.	pal	N, NE, CO	M&A, F, MJr	TDM Barbosa 1218 (INPA, UEC)
*Polygala appressa* Benth.	pal	N, NE	M&A, F, MJr	TDM Barbosa 1145 (INPA, UEC)
*Polygala longicaulis* Kunth	pal	N, NE, CO, SE, S	M&A, F, MJr	TDM Barbosa 1185 (INPA, UEC)
*Polygala trichosperma* Jacq.	pal	N, NE	F, MJr	SM Costa 954 (INPA)
*Polygala violacea* Aubl.	pal	N, NE, CO, SE, S	F	DM Cavalcanti 205 (INPA)
PONTEDERIACEAE (1/3)				
cf. Eichhornia crassipes (Mart.) Solms	ffloat	N, NE, CO, SE, S	F, MJr	-
*Eichhornia diversifolia* (Vahl) Urb.	emer, sub, fleav	N, NE, C, SE, S	M&A, F, MJr	TDM Barbosa 1187 (INPA, UEC)
*Eichhornia heterosperma* Alexander	emer, sub	N, NE, CO, SE	F	TDM Barbosa 1352 (INPA, UEC)
RAPATEACEAE (4/6)				
*Cephalostemon affinis* Körn.^+^	pal, emer	N, CO	F	TDM Barbosa 1256 (INPA, UEC)
*Duckea squarrosa* (Willd. ex Link) Maguire *	pal, emer	N	F	TDM Barbosa1273 (INPA, UEC)
*Monotrema aemulans* Körn.* ^+^	pal, emer	N, CO	F	SMCosta 882 (UEC)
*Monotrema bracteatum* Maguire ^& +^	pal, emer		F	TDM Barbosa 1281 (INPA, UEC)
*Monotrema xyridoides* Gleason ^+^	pal, emer	N	F	TDM Barbosa 1225 (INPA, UEC)
*Spathanthus bicolor* Ducke	pal, emer	N	F	TDM Barbosa 1297 (INPA, UEC)
RUBIACEAE (4/7)				
*Borreria alata* (Aubl.) DC.	pal	N, NE, CO, SE, S	F	TDM Barbosa 1190 (UEC)
*Borreria capitata* (Ruiz & Pav.) DC.	pal	N, NE, CO, SE, S	M&A, F	SM Costa 976 (UEC)
*Borreria verticillata* (L.) G.Mey.	pal	N, NE, CO, SE, S	M&A, F, MJr	SM Costa 976 (UEC), N Dávilla 6309 (INPA)
*Declieuxia fruticosa* (Willd. ex Roem. & Schult.) Kuntze	pal	N, NE, CO, SE, S	F	TDM BArbosa 1131 (INPA, UEC)
*Perama galioides* (Kunth) Poir.	pal	N, CO	F	TDM Barbosa 1143 (INPA, UEC)
*Perama hirsuta* Aubl.	pal	N, NE, CO, SE	M&A, F	TDM Barbosa 1308 (UEC)
*Sipanea pratensis* Aubl.	pal	N, NE, CO, SE	M&A, F, MJr	TDM Barbosa 1178 (INPA, UEC)
SOLANACEAE (1/1)				
*Melananthus ulei* Carvalho^#^	pal	NE, CO	F	FN Cabral 392 (UEC)
VERBENACEAE (1/1)				
*Stachytarpheta angustifolia* (Mill.) Vahl *	pal	N, NE, SE	F, MJr	SM Costa 974 (INPA, UEC)
XYRIDACEAE (2/20)				
*Abolboda americana* (Aubl.) Lanj.	pal	N, NE, CO, SE	F	SM Costa 703 (INPA, UEC)
*Abolboda killipii* Lasser ^+^	pal	N	F	TDM Barbosa 1095 (INPA, UEC)
*Abolboda macrostachya* Spruce ex Malme	pal	N, CO	F	TDM Barbosa 1332 (INPA, UEC)
*Abolboda pulchella* Humb. & Bonpl.	pal	N, NE, CO, SE	F	TDM Barbosa 1346 (INPA, UEC)
*Xyris cryptantha* Maguire & L.B. Sm. ^+^	pal	N	F	SM Costa 704 (INPA, UEC)
*Xyris dilatatiscapa* Kral & Jans.-Jac. ^+ $^	pal	N		Mota et al. (2015)
*Xyris fallax* Malme	pal	N, NE, CO, SE	F	SM Costa 741 (INPA,UEC)
*Xyris guianensis* Steud.	pal	N	F	NFO Mota 2313 (INPA,UEC)
*Xyris involucrata* Nees	pal	N	F	Mota et al. (2015)
*Xyris jupicai* Rich.	pal	N, NE, CO, SE, S	F, MJr	SM Costa 884 (INPA,UEC)
*Xyris laxifolia* Mart. [=*Xyris macrocephala* Vahl]	pal	N, NE, CO, SE, S	M&A, F, MJr	Mota et al. (2015)
*Xyris malmeana* L.B.Sm.^+^	pal	N, NE, CO	F	SM Costa 778 (INPA,UEC)
*Xyris mima* L.B. Sm. & Downs	pal	N	F	TDM Barbosa 1442 (INPA,UEC)
*Xyris paraensis* Poepp. ex Kunth	pal	N, NE, CO	M&A, F, MJr	SM Costa 909 (INPA,UEC)
*Xyris savanensis* Miq.	pal	N, NE, CO, SE, S	M&A, F, MJr	SM Costa 784 (INPA,UEC)
*Xyris subglabrata* Malme	pal	N	F	TDM Barbosa 1217 (INPA,UEC)
*Xyris subuniflora* Malme	pal	N	F	TDM Barbosa 799 (INPA,UEC)
*Xyris surinamensis* Spreng.	pal	N	F	TDM Barbosa 1306 (INPA,UEC)
Xyris uleana var. angustifolia Lanj.	pal	N	F	SM Costa 753 (INPA)
*Xyris uleana var. uleana* Malme	pal	N, CO	F	SM Costa 800 (INPA)
*Xyris* sp.	pal	-		Mota et al. (2015)

## Results

**Figure 3.**
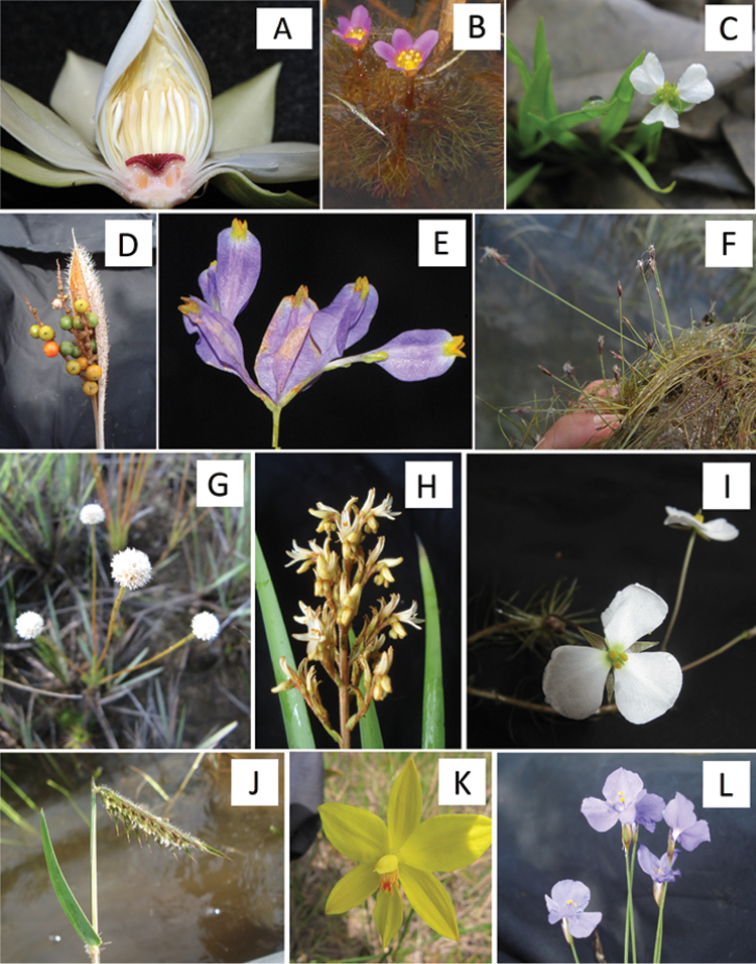
Wetland basal angiosperms and monocots of Viruá National Park (selected examples). **A** *Nymphaea amazonum* Mart. & Zucc. **B** *Cabomba furcata* Schult. & Schult. f. **C** *Helanthium tenellum* (Mart. ex Schult. & Schult. f.) Britton **D** *Bactris campestris* Poepp. **E** *Burmannia bicolor* Mart. **F** *Eleocharis fluctuans* (L.T. Eiten) E.H. Roalson & C.E.Hinchliff **G** *Syngonanthus fenestratus* Hensold **H** *Schiekia orinocensis* (Kunth) Meisn. **I** *Mayaca longipes* Mart. ex Seub. **J** *Echinolaena inflexa* (Poir.) Chase **K** *Duckeella pauciflora* Garay **L** *Abolboda pulchella* Humb. & Bonpl.

**Figure 4.**
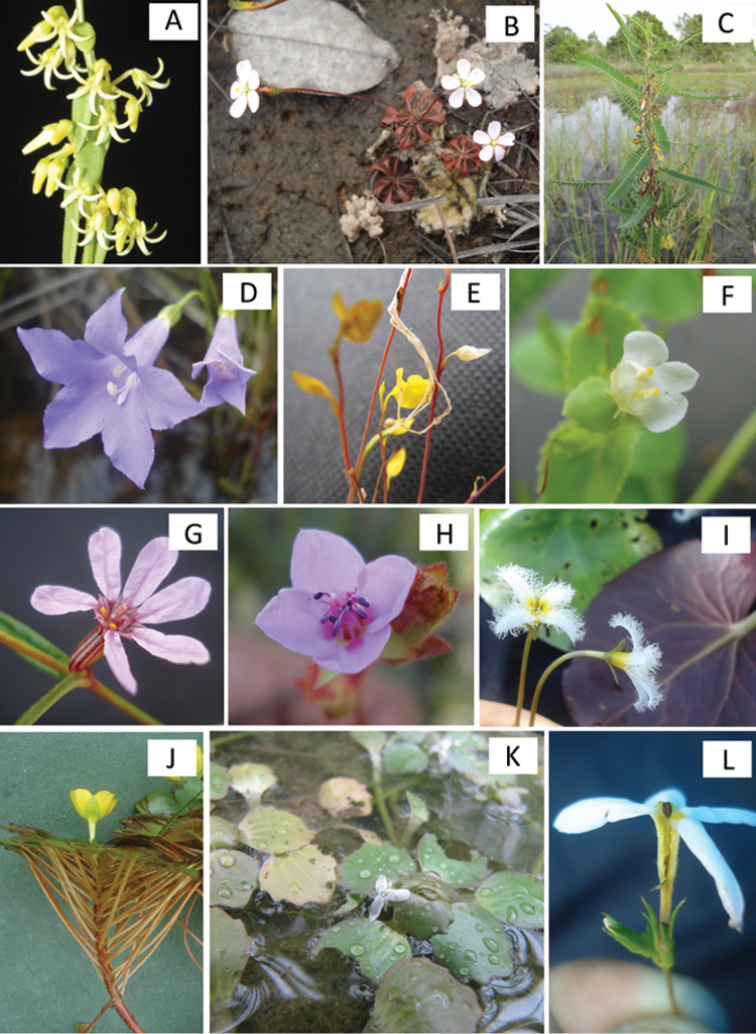
Wetland eudicots of Viruá National Park (selected examples). **A** *Cynanchum guanchezii* Morillo **B** *Drosera kaieteurensis* Brumm.-Ding. **C** *Aeschynomene scabra* G.Don **D** *Irlbachia pratensis* (Kunth) L.Cobb & Maas **E** *Utricularia chiribiquetensis* Fernandez-Pérez **F** *Lindernia diffusa* (L.) Wettst **G** Cuphea cf. gracilis Kunth **H** *Acisanthera tetraptera* (Cogn.) Gleason **I** *Nymphoides indica* (L.) Kuntze **J** *Ludwigia sedoides* (Humb. & Bonpl.) H.Hara **K** *Bacopa egensis* (Poepp.) Pennell **L** *Sipanea pratensis* Aubl.

The INPA herbarium was the only that held older specimens collected in the study area. Our final list includes 207 species of A&P herbs and subshrubs, distributed in 85 genera and 37 families (Table 1; Figures 3–4). Nine species remain identified only at genus level or at species level but with doubtful status (cf./aff.).

The richest families are Cyperaceae (45 spp.), Lentibulariaceae (25 spp.) and Xyridaceae (20 spp.) and the richest genera are *Utricularia* L. (Lentibulariaceae; 22 spp.), *Xyris* L. (Xyridaceae; 16 spp.) and *Rhynchospora* Vahl (Cyperaceae; 15 spp.).

We recorded six new occurrences and one probable new occurrence for Brazil, two new occurrences for the northern region and 21 new occurrences for the state of Roraima. Our list presents six new occurrences for Brazil, namely: *Rhynchospora maguireana* T. Koyama and *Scleria amazonica* Camelbeke, M.Strong & Goetgh. (Cyperaceae), *Drosera kaieteurensis* Brumm.-Ding. (Droseraceae), *Croton subserratus* Jabl. (Euphorbiaceae), *Siphanthera cowanii* Wurdack (Melastomataceae) and *Monotrema bracteatum* Maguire (Rapateaceae); and a probable new occurrence: Cuphea cf. gracilis Kunth (Lythraceae). Additionally, there are two new records for the northern Brazilian region (*Aeschynomene scabra* G.Don and *Eichhornia heterosperma* Alexander).

All life forms were registered in the VNP (see Table 1), with 20% of the species included in more than one category. Palustrine plants encompass 175 species (approximately 85% of species). The most species-rich families are Cyperaceae (41 spp.), Xyridaceae (21 spp.) and Lentibulariaceae (17 spp.), all of them common in waterlogged soils. The emergent and submerged categories presented 43 and 23 species, respectively, and eight species rooting in mud and with floating leaves; while among the free-floating plants solely two species were recorded (*Pistia stratiotes* L. and cf. *Eichhornia crassipes* (Mart.) Solms). Taking into account only the submerged plants, Lentibulariaceae was the most species-rich family, with eight species of *Utricularia*. Each of the other families with plants adapted to submersed conditions had between one and three species. Among the families with emergent plants, Cyperaceae was the most diverse, with 16 species distributed in seven genera.

As regards A&P angiosperms listed here, only 13 species appear to be restricted to white-sand savannas (see Table 1), most of these also recorded for areas in the Guiana Shield. Approximately 56 species occur solely in the northern region of Brazil, and five of them are only found in Roraima state.

## Discussion

### Identification of A&P plants occurring at VNP

On the identification of A&P plants occurring at VNP, it required the examination of several references, *online* collections and specialists. This correct identification is important as it allows the accurate list and comparison of subsampled vegetation (white-sand savannas; Vicentini 2004) and Amazon area (Hopkins 2007) with other areas and vegetation.

We still are studying some specimens of VNP, but especially when they belong to large genera, such as *Eleocharis* R.Br. (Cyperaceae), this process can take considerable time in the gathering of scattered and sometimes hardly accessible references.

We observed a population of cf. *Eichhornia crassipes* in the VNP but all individuals were sterile. The identification of sterile specimens is sometimes imprecise but floating islands of *Eichhornia crassipes* were observed in the Branco River (near the urban area of Caracaraí and out of the VNP) and the sterile population shown the aerenchymatous petiole characteristic of this species (Horn 2004). So we decided to list it as cf. *Eichhornia crassipes* (Table 1).

A peculiar case of complex identification in the VNP is the case of *Bacopa egensis* (Poepp.) Pennell (currently in Plantaginaceae). The identification to genus or even family was difficult, as the floras and taxonomical works consulted did not include the respective genus or contained errors in the identification keys and/or inaccurate descriptions. Not even the description provided by Souza and Giulietti (2009) encompass the morphological variation exhibited in the specimens of the VNP (K4 C3 A3). Specialists previously classified this species in the genus *Hydranthelium* Kunth and it does not fit in most descriptions of Scrophulariaceae s.l. or Plantaginaceae or even *Bacopa*.

### New occurrences and endemisms in white-sand savannas/“campinaranas”

Our data indicated new occurrences to both Roraima state and Brazil. It includes species previously registered for the Guiana Shield, an adjacent phytogeographical unit to the north (Funk et al. 2007).

Additionally, at least seven species among the new registers to Roraima are widely distributed in other Brazilian regions, e.g., *Syngonanthus cuyabensis* (Bong.) Giul., Hensold & L.R. Parra (Eriocaulaceae), *Utricularia gibba* L., *Utricularia hydrocarpa* Vahl, *Utricularia nana* A.St.-Hil. & Girard (Lentibulariaceae), *Nymphaea amazonum* Mart. & Zucc. (Nymphaeaceae), *Andropogon virgatus* Desv. and *Axonopus fissifolius* (Raddi) Kuhlm. (Poaceae). The two new records to northern Brazil, *Aeschynomene scabra* and *Eichhornia heterosperma*, are widely distributed species (Amaral 2013, Lima and Oliveira 2013) that simultaneously occur in the Guiana Shield and other Brazilian regions (Funk et al. 2007, Forzza et al. 2015). The delay of their register may be caused by the insufficient collection effort in Amazonia region.

Among A&P plants, only a few *taxa* are unique to a vegetation type and/or present restricted distributions (Sculthorpe 1967), and rare species generally display larger distribution areas than those of rare terrestrial species (Santamaría 2002); although Podostemaceae is an exception worth mentioning as they are commonly endemic to a single basin or even a single waterfall (Cook and Rutishauser 2007). Endemic species may occur in white-sand savannas, the predominant vegetation type in the VNP, and such species are probably spread in the numerous isolated “islands” of this vegetation type within rainforest (Anderson 1981).

There is no species endemic to the VNP and the endemic species of white-sand savannas belonging to the A&P plants that we listed here are not restrict to Brazilian territory and occur in other northern South American countries (such as Venezuela, Guyana and/or Colombia) (Table 1). Additionally, in Brazil the apparent restricted distribution in Roraima state may be in part the result of insufficient collections in white-sand savannas. We identified some specimens collected in this phytophysiognomies in Amazonas state and not yet deposited in any herbaria, which belong to such endemic species. In fact, endemic species of white-sand savannas were recently listed in other studies of the Viruá National Park (Cabral 2011, Dávila 2011, Azambuja 2012, Cangani 2012, Pessoa et al. 2015, Mota et al. 2015). Other *taxa* with a more restricted distribution are families, genera or species with concentrated richness or exclusive occurrence in northern South America, or only in the Guiana Shield, e.g. Rapateaceae, *Abolboda* Bonpl. and *Utricularia benjaminiana* Oliv.

### Life forms

We registered in the VNP all life forms usually recognized to these plants (Cronk and Fenessy 2001, Chambers et al. 2008), namely: palustrine (e.g. Xyridaceae), emergent (e.g. *Montrichardia arborescens*), with floating leaves (e.g. *Nymphaeae* spp.), free floating (e.g. *Pistia stratioides*) and submerged (e.g. *Mayaca* spp.). It is known that the number of species decreases towards strictly submerged plants (Barrett et al. 1993), as observed in our data.

Gribel et al. (unpublished data) reported the occurrence of species belonging to two genera of free floating plants in the “*buritizais*” in the study area (*Wolffia* Horkel ex Schleid. and *Spirodela* Schleid.), but we did not find specimens of these genera during our expeditions or in herbaria we consulted. Free-floating species depend on the nutrients dissolved in the water column (Cronk and Fenessy 2001) and the low richness at VNP may be due to the *igapó* characteristics attributed to the local waterbodies (low inorganic nutrient concentrations) (ICMBio 2014, Junk et al. 2011).

### Floristic connections

Concerning to the floristic comparisons, 189 species listed here were also found in Funk et al. (2007); 69 species in Moura-Júnior et al. (2015); 50 species in Miranda and Absy (1997); 13 species in Conserva et al. (2008); nine in Junk and Piedade (1993) and four in Piedade et al. (2010) (Table 1).

There are relatively few floristic lists for wetland plants in the Amazon region (Piedade et al. 2010) and in particular the northern Brazilian region remains strongly undercollected (Hopkins 2007, Schulman et al. 2007, Piedade et al. 2010). This situation makes it difficult or even impossible for us to compare our results with other works and probably turns artifacts caused by low sampling into apparent patterns (Hopkins 2007, Piedade et al. 2010).

Another study focusing on A&P plants also carried out in the VNP is yet unpublished (Paiva 2012). In it, the collecting effort was concentrated in the PPBio (Biodiversity Research Program) grid, in a more forested area within the national park, and in an area of hydromorphic open white-sand savanna near the “Estrada Perdida” road. The authors listed 19 species, some of them present in our list. The material was identified only in part and at genus level and at the time of our visit to the herbaria in Roraima, the vouchers of Paiva (2012) were not available yet. Since we could not analyze those specimens, the different taxa there listed do not appear in our list, so further comparisons could not be made.

The similarity of the aquatic and palustrine flora of the Viruá National Park with that of the Guiana Shield is evident, only 17 species of our list are absent in Funk et al. (2007) list. One must consider the geographic proximity between the two areas. Additionally, both have common limiting conditions for many plant species, mainly the nutrient-poor soils (Janzen 1974, Prance and Schubart 1978, Anderson 1981, Steyermark et al. 1995, Funk et al. 2007).

Junk et al. (2011) classified the rivers of the study area, including the sediment-rich Branco River, as black- or clear-water due to the low levels of dissolved nutrients. The *igapó* and *várzea* differ regarding their floristic composition and as to their ecological patterns (Prance 1979, Klinge 1983, Pires and Prance 1985, Piedade et al. 2010, Junk et al. 2011). Prance (1979) refers to the exclusive occurrence of some species to each vegetation type: e.g. *Bombax munguba* Mart. (Malvaceae), *Couroupita subsessilis* Pilg. (Lecythidaceae) and *Hevea brasiliensis* Muell. (Euphorbiaceae) to *várzea* areas; and *Couepia paraensis* (Mart. & Zucc.) Benth. and *Licania apetala* (E. Mey.) Fritsch (Chrysobalanaceae) and *Tabebuia barbata* (E. Mey.) Sandw. at igapó areas.

Two species reported in our list, namely *Bacopa egensis* (otherwise collected in the Solimões River, in Central America – including in rice-fields – and even in swamps near New Orleans, Louisiana; Christenhusz 2014) and *Glinus radiatus* (Ruiz & Pav.) Rohr. (sometimes recorded as weed) are supposedly associated with nutrient-rich habitats. These species were only collected in a lake less than 1 km from the margin of the Branco River (*Ano Bom* lake, which receives floodwater from the Branco River periodically), in an area with gray clay soil. Junk et al. (2011) state that the water of the Branco River, which resembles a white-water river, has low nutrient status and it is thus chemically closer to a clear-water river. The presence of these two species suggests that the Branco River may be richer in nutrients than a clear-water river.

The comparison of our list with that of an area of *várzea* near Manaus (Junk and Piedade 1993) illustrates some of these differences between nonarboreal *Várzea* and *Igapó* areas; only a few species are common to both areas and in the *várzea* vegetation Poaceae and Araceae s.l. (predominantly Lemnoideae) are the richest families of wetland species. Another study of wetland plants at the Amazonas/Solimões River interface also revealed a low number of species in common with the VNP (Conserva et al. 2008). However, both lists (Junk and Piedade 1993, Conserva et al. 2008) are based on expeditions made during the terrestrial phase (dry season), to facilitate access to the localities, influencing the results. Thus, there is an increasing richness of weedy species not necessarily adapted to wetland conditions in both inventories.

Few studies have been carried out in areas of *igapó* that focus on the diversity of herbaceous and subshrubby aquatic and palustrine plants (Piedade et al. 2010). Lopes et al. (2014) present a floristic analysis of A&P plant genera in six river systems with igapó characteristics in the Amazonas state. They listed 25 families and 63 genera, from which 15 families and 24 genera occur in the Viruá National Park (Lopes et al. 2014). Similarly to the pattern observed by Lopes et al. (2014), Cyperaceae is richer than Poaceae in the VNP. Those authors did not provide a species list and comparisons are not possible.

Indirectly, Piedade et al. (2010) mention four species to igapó areas: *Oryza perennis* Moench, *Nymphaea rudgeana* G. Mey., *Utricularia foliosa* L. and *Cabomba aquatica* Aubl. As regards the yellow-flowered *Cabomba* Aubl. with four tepals that occurs in the VNP and in the Jufari River (see Piedade et al. 2010), we identified this species as *Cabomba schwartzii* Rataj. Currently, specialists list this species as a synonym of *Cabomba aquatica*, typically with six tepals (Forzza 2015). The name *Oryza perennis* is doubtful and specialists will soon submit a proposal to reject it (Robert Soreng, pers. comm.). The specimens named *Oryza perennis* in Amazonia and the Guianas are probably *Oryza rufipogon* Griff. Therefore, we also recorded all four species mentioned by Piedade et al. (2010) for the Jufari River in the VNP; all are widely distributed species.

At northern Roraima there is other non-forest vegetation with different abiotic conditions enclosed in the matrix of Amazonian rainforest, the savannas (ICMBio 2014; IBGE 2012). Again, we know little regarding A&P plants in the savannas of Roraima and must take some care when making comparisons with the few works published, such as considering the need for a revision of the identifications published in older lists. The limitations of useful taxonomic literature, faced especially before the publication of the Flora of the Venezuelan Guayana, were considerable and sometimes researchers vaguely delimited study areas.

The floristic survey published by Miranda and Absy (1997) gathers data from various previously published inventories and results of the author’s collections in the savanna region of Roraima, being the most complete list available to this area. It contains nearly 300 species: c. 30% are from wetlands and of these, 1% in strictly aquatic habitats. We herein recorded 50 of these species for the Viruá National Park, mostly species with a large geographical distribution. More recent lists of aquatic and palustrine species from Roraima state are unpublished (Neves 2007, Paiva 2012), thus only presenting a low number of more widely distributed species.

In the updated checklist of macrophytes from northern Brazil, that is based on previous lists plus recent data collected by the authors and data contained in the SpeciesLink and “*Lista da Flora do Brasil*” platforms, Júnior et al. (2015) listed about 540 spp. of A&P plants, among those species only 68 are in common with our list (Table 1). As their list probably gathers information from *Várzea* and *Igapó* areas with no distinction, useful discussions cannot be made at this moment. Anyway, some care must be taken as their list was based on information of the SpeciesLink platform, (according to Moura Júnior et al. 2015), where not all specimens are correctly identified. The criterion for the inclusion of the species used by those authors is not clear. Similarly, though the “*Lista da Flora do Brasil*” is a great source of information about species of the Brazilian flora (as cited by Moura Júnior et al. 2015), it is still incomplete and with considerable data gaps for some taxa and geographical regions, such as aquatic and palustrine species and northern Brazil.

### Conclusions and future studies

Despite the need for more collecting effort in the inner parts of the VNP, its flora of aquatic and palustrine herbaceous and subshrubby angiosperms is clearly connected to the flora of the Guiana Shield, an adjacent phytogeographical region to the north and geologically related to the origin of white-sand savannas, the predominant physiognomy in the protected area studied here.

Only the INPA herbarium held old specimens from the studied area and although we found some bibliography concerning wetland plants of the region, publications are scattered or the most complete refer mainly the Venezuelan and/or Guiana territory. A greater collecting effort and the revision of herbarium specimens are essential to allow for a meaningful evaluation of the similarities and differences between the white-sand savannas and other savanna areas of the Amazon. Reasonably complete lists of aquatic and palustrine plants in areas influenced by *igapó* rivers, white-sand savannas and other savannas in the Amazon region may also uncover floristic, biogeographical, evolutionary and ecological patterns currently obscured by the inadequate collection status.

To allow for the identification of the species here listed, our group is currently producing keys (including interactive multi-access keys), descriptions with images, taxonomic comments, geographical distribution and field observations of the listed *taxa*. We will provide these resources shortly, in the format of an eFlora on a website about the Viruá National Park (Costa et al. in prep.).
